# Factors Affecting Middle School Teachers’ Attitudes Toward the Inclusion of Students With Disabilities

**DOI:** 10.3389/fpsyg.2022.853696

**Published:** 2022-03-23

**Authors:** Mubarak S. Aldosari

**Affiliations:** Special Education Department, Prince Sattam Bin Abdulaziz University, Al-Kharj, Saudi Arabia

**Keywords:** inclusion, attitudes, ORI, Saudi Arabia, teacher, students with disabilities, middle school

## Abstract

Teachers’ positive attitudes are an essential element for the successful inclusion of students who have disabilities in schools with their peers who do not have disabilities. The current quantitative study examines middle school teachers’ attitudes toward the inclusion of students with disabilities in regular schools in Saudi Arabia and the factors that affect their attitudes. Middle school teachers (*N* = 613) from schools in Riyadh responded to a questionnaire regarding their opinions relative to the integration of students with disabilities. The results indicate that middle school teachers have a neutral attitude toward the inclusion of students with disabilities in regular schools. Moreover, teacher-related factors such as gender, position, and experience do not affect teacher attitudes toward inclusion. In contrast, training in inclusion plays a significant role in middle school teachers’ attitudes toward inclusion of students with disabilities. The implications of the results and suggestions for further research are discussed in the study.

## Introduction

Many nations across the globe have begun to consider the best practices for educating students with disabilities. One such example is Saudi Arabia, which has witnessed a greater development of special education over the last two decades. Approximately 96% of students with profound disabilities in Saudi Arabia were admitted to separate special education schools during the 2007–2008 academic year. About 88% of students with mild disabilities such as learning disabilities, however, were integrated into regular classrooms under the inclusion model (Ministry of Education of Saudi Arabia, 2008). Saudi Arabia has continued to utilize a model of inclusion in its education system over the years, and many other nations focus on using a similar model.

Inclusive education has several benefits as it increases sensitivity, empathy (by reducing the prevalence of bullying of students with disabilities), social development, and academic achievement. Studies done in Australia, Ireland, Russia, and elsewhere show that it reduces the segregation of students based on disabilities (Muniroh et al., 2017; Anderson and Boyle, 2019; Kamenez et al., 2019; King and Ryan, 2019; Kurilenko et al., 2020). Additionally, a study done in the United States observed a positive relationship between the rate of inclusion of students with disabilities and academic achievement of students with and without disabilities (Hunt, 2000).

The success of the inclusion process depends on various factors such as the qualification and ability of the teachers (Toomsalu et al., 2019; Ovcharenko et al., 2021) and their attitudes toward the inclusion of students with disabilities (Leyser and Tappendorf, 2001). Ovcharenko et al. (2021) found that prospective music teachers in Ukraine who received pedagogical instruction targeted toward teaching music in an inclusive setting were able to improve educational outcomes for their students more than teachers who had not received the instruction. Opertti and Brady (2011) and Carrington and MacArthur (2012) concluded that teachers’ attitudes play a vital role in successful inclusion. Similarly, Anderson and Boyle (2015) noted that negative teacher attitudes hinder the success of inclusion. Therefore, these studies appear to show that teachers’ attitudes are a crucial part of successful inclusive education.

Although several more studies have examined the impact of teachers’ attitudes and associated factors on inclusive education, they have yielded inconsistent results. Some studies indicate that factors such as teachers’ gender, education, experience, and training do not influence inclusion education. However, other studies have found a direct association between these factors and inclusion. For instance, Rana (2012) and Emmers et al. (2020) found no relationship between teachers’ attitudes and gender. In contrast, Alahmadi (2009) found that female teachers showed less positive attitudes toward inclusion in Saudi Arabia, and Sharma et al. (2012) found similar study results in India. However, Forlin et al. (2009)’s study done with participants in Australia, Canada, Hong Kong, and Singapore and Kuyini and Mangope’s (2011) study done in Ghana and Botswana observed that male teachers showed less positive attitudes than female teachers.

Some research indicates that teachers with relatively greater professional experience are more supportive of inclusive education compared with those who have lesser experience, owing to greater confidence and knowledge (Al-faiz, 2006; Ernst and Rogers, 2009; Rakap and Kaczmarek, 2010). However, Parasuram (2006) and Sharma et al. (2008) concluded that professional teachers with fewer than five years of work experience support inclusion and show relatively greater positive attitudes than teachers with more experience.

Training is another important factor that can influence an inclusion-based education model. While some studies report that teachers with inclusion training perform better than those with no training (Romi and Leyser, 2006; Avramidis and Kalyva, 2007), others report that training is independent of teachers’ attitudes toward the inclusion of students with disabilities (Alquraini, 2012).

Another primary factor is the current teaching position. Many studies have found that special education teachers show more positive attitudes toward inclusion than general education teachers do (Lifshitz et al., 2004; Cameron, 2014). A survey done of in-service teachers attending a U.S state university in the mid-west found that the special education teachers were more likely to have the attitude that a general classroom environment could serve the needs of all students, while the general education teachers were more likely to have the attitude that educating a student with a disability would have a negative effect on the typical students in the classroom (Elhoweris and Alsheikh, 2006).

It is also important to consider the personal attitude of teachers toward using the inclusion model and the implications it may have on their practice. Multiple international studies have examined teacher attitudes toward inclusion. Neal (2016) conducted a study in the United States that focused on the attitudes of elementary, middle, and high school teachers and administrators in a southeastern school district. In addition to the relationship between teacher training and teacher attitudes toward inclusion, the study found overall positive attitudes toward inclusion education. It also found that the more positive attitude a participant had toward special education, the less restrictive environment for students the participant preferred. Another study was conducted in France by Jury et al. (2021) that examined teachers’ attitudes toward including students with autism spectrum disorder (ASD) in mainstream classrooms. It gathered data on middle and high school teachers’ perceptions and found overall positive attitudes toward inclusions. It also discovered that the study participants had a more positive attitude toward having students they perceived to have “no difficulties” in the classroom as opposed to students with behavioral or cognitive difficulties.

In Saudi Arabia as well, many studies have investigated the viewpoints of general and special education teachers regarding inclusive education in public elementary schools. For instance, Alahmadi (2009) reported that both types of teachers showed negative attitudes toward the inclusion of students with disabilities. Similarly, Alquraini (2012) observed teachers’ perspectives regarding the inclusion of students with intellectual disabilities in a general elementary school. The results indicated that teachers held slightly negative perspectives toward inclusion. Alasim and Paul (2019) also explored the attitudes of special education teachers and general education teachers toward the inclusion of deaf or hard of hearing students in public elementary schools in Saudi Arabia. Their findings showed impartial attitudes from both types of teachers. Abaoud (2013) conducted a study determining teachers’ willingness to teach students with attention deficit hyperactivity disorder and concluded that elementary school teachers in Riyadh had a neutral attitude toward students with this condition.

Analysis of the Saudi literature examining educators’ attitudes toward the inclusion of students with disabilities in regular classrooms indicates that the focus is generally on elementary school teachers. The attitudes of middle and secondary school teachers, therefore, have not been sufficiently explored. Alquraini (2012) and Alasim and Paul (2019) also suggested a future investigation into middle school teachers’ attitudes toward the inclusion of students with disabilities.

To fill this literature gap, the current study has the following objectives: 1) to examine middle school teachers’ attitudes toward the inclusion of students with disabilities in regular schools and 2) to examine the relationship between teachers’ attitudes toward the inclusion of students with disabilities and factors such as teachers’ education, gender, experience, and training in inclusion. This study is significant as teachers’ attitudes can affect teaching strategies, curriculum considerations, teachers’ professional development, and communication between typical students and students with disabilities (Logan and Wimer, 2013).

## Methods

### Participants

The participants were teachers employed in government middle schools in Riyadh that included special education programs during the 2021–2022 academic year. The city has 59 government middle schools that include special education programs. These schools have 3,333 students with disabilities, 450 special education teachers, and 2,160 regular teachers (Ministry of Education, 2021). The researcher chose to include the teachers in all 59 middle schools.

### Instrument

Quantitative data were collected through the opinions relative to integration of students with disabilities (ORI) questionnaire (Antonak and Larrivee, 1995). In 1977, Cook and Larrivee developed the initial version of the ORI to assess teachers’ attitudes toward the inclusion of students with disabilities. This initial version was called “opinions relative to mainstreaming” and it included 30 items scored on a 5-point Likert scale. In 1995, the two authors revised the instrument and renamed it the ORI.

The ORI includes 25 items scored on a 6-point Likert scale. It includes four dimensions: (1) benefits of integration, (2) integrated classroom management, (3) perceived ability to teach students with disabilities, and (4) special vs. integrated general education teachers.

Participants also responded to questions pertaining to demographic characteristics: (1) teaching experience, (2) gender, (3) current teaching position (general or special education), and (4) training in inclusion.

The researcher used ORI to measure the perspectives of teachers regarding the inclusion of students with disabilities in this study for several reasons. First, the ORI has comprehensive factors that can be used to gather adequate information regarding teachers’ perspectives toward the inclusion of students with disabilities. Second, the contents of the items are especially appropriate for obtaining information regarding the background of special education and general education teachers in Saudi Arabia. For example, these items do not include advanced concepts of inclusion, such as modifications and adaptations of the general curriculum or use of assistive technology, as these issues are likely to be unfamiliar for these teachers (Alquraini, 2011).

In addition, previous studies that used the ORI scale in different countries and different languages were able to demonstrate that the ORI scale is a reliable and valid instrument (e.g., Cook, 2002; Dupoux et al., 2006; Alasim and Paul, 2019). As cited in Antonak and Larrivee (1995), the ORI has also been used in the past to investigate the attitudes of teachers teaching grades kindergarten through 12. Since the topic of this study is the attitudes of middle school teachers, the ORI is an appropriate tool to use.

#### Translation of the Opinions Relative to Integration of Students With Disabilities

Richard Antonak provided authorization to utilize the ORI in a momentum study. The ORI was translated into Arabic by bilingual experts from the English Department at Prince Sattam bin Abdul-Aziz University. Subsequently, the questionnaire was translated back into English without comparing it to the actual English version. This was done to guarantee the validity and reliability of the questionnaire.

#### Pilot Study

A pilot study was conducted to determine the practicality of utilizing the Arabic version of the ORI for this study. Specifically, the goal was to determine the adequacy of the language and length of the questionnaire. The questionnaire’s social pertinence was also examined, and if the respondents offered any appropriate suggestions, these were incorporated into the final version.

Before administering the Arabic version of the ORI, the researcher received approval from the Institutional Review Board of Prince Sattam Bin Abdulaziz University and the Ministry of Education. Forty-three out of 50 teachers responded to the online ORI (Google Forms survey) in the pilot study.

The researcher ensured that the pilot study included a sample similar to the main study, that is, special education and general education teachers working in middle schools that have special education programs. Through the statistical analysis, it was found that Cronbach’s alpha for the ORI was 0.70. Although most ORI items were positively correlated with the total score, items 1, 13, and 25 were negatively correlated with the total score (−0.43, −0.01, 0.023, respectively).

Furthermore, a few teachers indicated that items 17 and 21 needed to be explained because they were intricate and not expressed clearly. Therefore, the researcher carefully revised these items for clarity. These procedures improved the language, content, and validity of the final Arabic version of the ORI.

### Procedures

Before administering the ORI, the researcher received approval from the Ministry of Education to conduct the study and obtained the contact details of the principals of the 59 middle schools from the Ministry of Education.

The principals were asked to send the Google Forms questionnaire link to all teachers in the 59 schools. The participants were asked to respond to the questionnaire after providing written informed consent. In approximately two weeks, 644 teachers submitted the completed surveys; of these, 31 were excluded because of missing data. Therefore, the final sample consisted of 613 teachers.

## Data Analysis

Table 1 summarizes the participants’ demographic information. Of the participating teachers, 370 (60.4%) were men and 243 (39.6%) were women. In addition, out of 613 teachers, only 131 (21.4%) had received training in inclusive education while 482 (78.6%) had not received any training. Furthermore, 352 (57.4%) participants were special education teachers and 262 (42.6%) were general education teachers. Moreover, only 39 (6.4%) participants had less than five years of teaching experience, 323 (57.2%) had less than 10, and 251 (40.9%) had more than 10.

**TABLE 1:** Demographic characteristics of middle school teachers.

Variables		Frequency	%
Gender	Men	370	60.4
	Women	243	39.6
Training in inclusive education	Yes	131	21.4
	No	482	78.6
Current teaching status	Special education	352	57.4
	General education	261	42.6
Teaching experience (years)	1–<5	39	6.4
	5–<10	323	52.7
	≥10	251	40.9

The average mean scores and their interpretation for teachers’ attitudes toward the inclusion of students with disabilities on the ORI are illustrated by Table 2.

**TABLE 2:** Interpretation of average mean ORI scores of attitudes toward inclusion.

Scale	Score range	Mean range
1	72.00–72.86	Disagree very much
2	72.86–73.71	Disagree pretty much
3	73.71–74.57	Disagree a little
4	74.57–75.43	Neutral
5	75.43–76.29	Agree a little
6	76.29–77.14	Agree pretty much
7	77.14–78.00	Agree very much

*ORI, Opinions Relative to Integration of Students with Disabilities.*

Analyzation of the data resulted in a mean of teacher attitudes toward inclusion of 75.38, as shown in Table 3. By relating this mean (75.38) with the interpretation given in Table 2, it can be observed that the mean ORI score of middle school teachers toward inclusion is in the fourth category (74.57–75.43). This indicates that middle school teachers presented neutral attitudes toward the inclusion of students with disabilities.

**TABLE 3:** Attitudes of middle school teachers toward inclusion.

	N	Mean	Standard deviation
Score	613	75.3817	17.66337
			

A one-way analysis of variance (ANOVA) was performed and independent two samples *t*-tests were used to analyze the factors associated with middle school teachers’ attitudes toward the inclusion of students with disabilities. The assumptions of homogeneity of variance and normal distribution were met for both statistical analyses. An independent two samples *t*-test was performed to compare attitudes toward the inclusion of students with disabilities in participants with male and female genders. Table 4 shows the results of the analysis.

**TABLE 4:** *T*-test: Differences among the participants based on gender.

Gender	n	Mean	Standard deviation	*T*-value	Sig
Men	370	75.7378	17.44582	0.616	0.538
Women	243	74.8395	18.01225		

The results revealed that there were no statistically significant differences in participants’ attitudes toward the inclusion of students with disabilities in teachers of the male gender (M = 75. 74, SD = 17.45) and teachers of the female gender (M = 74.84, SD = 18.01); (t (611) = 0.616, p = 0.538). The average attitudes of male participants were similar to those of female participants.

A one-way ANOVA was performed to compare the effects of teaching experience on middle school teachers’ attitudes toward the inclusion of students with disabilities. Table 5 shows the results of the analysis.

**TABLE 5:** ANOVA: differences among the participants based on teaching experience.

Source	Sum of squares	df	Mean square	*F*−value	Sig
Between groups	138555.614	2	69277.807	806.708	0.000
Within groups	52385.061	610	85.877		
Total	190940.675	612			

The results showed statistically significant differences in participants’ attitudes toward the inclusion of students with disabilities based on teaching experience [*F*(2, 610) = 806.708, *p* < 0.05]. This indicates that participants’ average attitudes differed across levels of teaching experience. The Scheffe multiple comparison tests showed that the attitude score of participants with under five years of teaching experience was significantly (p < 0.05) different from that of participants with under 10 years of experience and participants with more than 10 years of experience (Table 6).

**TABLE 6:** Scheffe: Multiple comparison test based on teaching experience.

Scheffe						

(I) Teaching experience (years)		Mean difference (I-J)	Standard error	Sig.	95% confidence interval	
					Lower bound	Upper bound
<5	5–<10	17.31762*	1.57094	0.000	13.4629	21.1723
	≥10	44.85187*	1.59503	0.000	40.9380	48.7657
5–<10	<5	−17.31762*	1.57094	0.000	−21.1723	−13.4629
	≥10	27.53426*	0.77975	0.000	25.6209	29.4476
≥10	<5	−44.85187*	1.59503	0.000	−48.7657	−40.9380
	5–<10	−27.53426*	0.77975	0.000	−29.4476	−25.6209

*The mean difference is significant at the 0.05 level.

An independent two samples *t*-test was conducted to compare attitudes toward the inclusion of students with disabilities in participants who are general education teachers and participants who are special education teachers. Table 7 shows that there were statistically significant differences in participants’ attitudes based on those who had inclusion training (*M* = 81.08, *SD* = 17.66) and those without inclusion training (M = 73.83, SD = 17.36); (*t* (611) = 4.218, *p* < 0.05). The average positive attitudes were significantly higher for participants with inclusion training compared with those without inclusion training.

**TABLE 7:** *T*-test: differences among the participants based on training for inclusion.

Training in inclusion	n	Mean	Standard deviation	*T*-value	Sig
Yes	131	81.0763	17.66423	4.218	0.000
No	482	73.8340	17.36095		

An independent two samples *t*-test was conducted to compare attitudes toward the inclusion of students with disabilities in participants who are general education teachers and participants who are special education teachers. Table 8 shows no statistically significant differences in attitudes based on those who are special education teachers (*M* = 75.84, *SD* = 17.18) and those who are general education teachers (*M* = 74.77, *SD* = 18.32); (*t* (611) = 0.743, *p* = 0.458). This indicated that current teaching positions did not influence participants’ attitudes toward inclusion.

**TABLE 8:** *T*-test: differences among the participants based on current teaching position.

Education area	n	Mean	Standard deviation	*T*-value	Sig
Special education	352	75.8381	17.17546	0.743	0.458
General education	261	74.7663	18.31611		

## Discussion

This study explored the attitudes of middle school teachers in Riyadh, Saudi Arabia toward the inclusion of students with disabilities. The results indicate that middle school teachers have a neutral attitude toward the inclusion of students with disabilities in mainstream schools. This finding is consistent with the results reported by Abaoud (2013) and Alasim and Paul (2019). It’s possible that teachers have a more neutral than positive attitude due to the nature of inclusion education in Saudi Arabia. An individualized education program is required for students with disabilities to ensure that each student receives personalized attention according to their needs, and this sometimes increases the burden on teachers as they attempt to provide equal attention to all students. This study also identified certain other factors that may influence the attitudes of middle school teachers toward the inclusion of students with disabilities.

The results revealed no relationship between teachers’ attitudes toward the inclusion of students with disabilities and teachers’ gender. This is consistent with studies by Rana (2012) and Emmers et al. (2020), who found no link between gender and teachers’ attitudes.

Research indicates that special education teachers have a more positive attitude toward the inclusion of students with disabilities than general education teachers (Lifshitz et al., 2004; Cameron, 2014). However, in the current study, there was no relationship between attitudes toward the inclusion of students with disabilities and current teaching positions. This is perhaps because most of the teachers (both special education teachers and regular teachers) who participated in the current study held bachelor’s degrees and were graduates of Saudi universities, which have similar programs for preparing teachers regardless of their specialization.

This study indicates that teachers with sufficient training regarding the inclusion of students with disabilities show more positive attitudes than those with no training. This finding is consistent with the results reported by Romi and Leyser (2006) and Avramidis and Kalyva (2007). This may be because inclusive education training provides greater subject knowledge regarding inclusive environments and exposure to diverse teaching approaches (Munoz and Chang, 2007). As deBettencourt (1999) noted, middle school teachers who had taken more special education courses than their peers were able to implement more special education strategies within the classroom. When teachers receive specific training, they are able to obtain a better understanding of the subject matter by using a variety of successful instructional tactics. They may discover or believe that they can satisfy the requirements of all students, regardless of their disabilities. This idea may contribute to a more favorable attitude toward students with special needs.

Previous studies have reported a positive correlation between teaching experience and teachers’ attitudes toward the inclusion of students with disabilities (Al-faiz, 2006; Ernst and Rogers, 2009; Rakap and Kaczmarek, 2010). However, in the current study, there was a negative correlation between teaching experience and attitudes toward the inclusion of students with disabilities—teachers with more teaching experience had more negative attitudes toward inclusion. One possible explanation is that experienced teachers are more aware of the challenges and difficulties of teaching students with disabilities. Some teachers may hold the belief that students with disabilities tend to have more difficult behaviors, and teachers with more experience in the classroom may not feel confident in their abilities to manage classroom behavior (Savolainen et al., 2012). On the contrary, teachers with less teaching experience and more recent formal training may have received more classroom management training and thus feel a greater sense of self-efficacy (Savolainen et al., 2012).

This study has certain limitations. First, it was conducted in Riyadh, Saudi Arabia. However, the country has many institutions and programs in different cities for children with disabilities. The results might have been different if the geographical area had been larger and non-government schools were included. Second, only quantitative data, which may not sufficiently explain attitudes, were used. The use of qualitative data may have proven helpful in presenting the findings of this study. It may have provided additional insights, as the research topic is of a subjective nature. In addition, the study’s findings on the relationship between current teaching positions and teacher attitude appears to contradict the findings on the relationship between inclusion training and teacher attitudes; further study is recommended to investigate any possible explanations. Finally, this study only included four factors associated with middle school teachers’ attitudes toward the inclusion of students with disabilities in mainstream schools. Future studies should include more factors associated with teachers’ attitudes.

## Conclusion

The current study bridges the research gap by examining the attitudes of middle school teachers toward inclusive education in Saudi Arabia, revealing relatively neutral attitudes. In particular, this study did not reveal any relationship between teachers’ gender and attitudes toward inclusion. However, training in inclusive education and teaching experiences were found to have significant effects on middle school teachers’ attitudes toward the inclusion of students with disabilities.

With the increasing diversity and numbers of students with special needs being served in general education classrooms, teachers need more knowledge about this population’s capabilities and more of the skills training that has been shown to be effective when working with them. Perhaps teachers’ attitudes will change if they acquire more knowledge and skills. Studies done in Europe, the United States, Asia, and Australia consistently show that teacher competence is a pre-requisite for successful inclusion, and higher levels of confidence are generally associated with positive teacher attitudes (Pit-ten Cate et al., 2018). It is a continuous challenge for teacher preparation programs to rethink practices and revamp strategies in order to prepare future general educators with the skills they require to meet the needs of diverse student bodies. Therefore, it is suggested that inclusive education courses be made mandatory in universities for all candidate teachers and that regular requisite in-service training be provided in schools. In addition, to ensure the success of inclusive education programs, emphasis on personnel and material support services with positive attitudes is essential.

This study found that teachers who had sufficient training in inclusion had a more positive attitude than those with no training, which is in line with the body of research that supports the idea that special education teachers tend to hold more positive beliefs about the potential success of students operating in the inclusion model (Lifshitz et al., 2004; Cameron, 2014). Therefore, it is suggested that experienced special education teachers lead some in-service trainings for their general education peers. One-on-one consultations between special education teachers and general education teachers may also be useful (deBettencourt, 1999).

More specifically, it is recommended to offer teacher training that focuses on the theory of metacognition. Drigas and Mitsea (2021) have created a model of metacognition training that may help teachers change their attitudes toward students with disabilities. Teachers who train their “visualization and scientific thinking skills (e.g., evidence-based research, group discussions, and evaluations of arguments)” (Drigas and Mitsea, 2020, p. 125) and strive toward evaluating their own prejudices and defense mechanisms should be able to have a more positive attitude toward teaching students with disabilities.

Metacognition training has also been shown to be enormously beneficial for students with disabilities (Hessels et al., 2009). Thus, there is potential for teachers to share their knowledge of the metacognition strategies they learn with their students. It is recommended for teacher training on metacognition to include practical methods for teachers to use to create targeted interventions focusing on problem-solving strategies, visualizing and organizing information, and future planning, etc. These strategies may be a way for students with disabilities to increase academic performance (Hessels et al., 2009) and access the higher levels of esteem and self-actualization found in Maslow’s Hierarchy (Maslow, 1987).

These additional trainings can be enhanced by offering a high level of social support to teachers. This support can be provided in different ways (emotional, informational, instrumental, etc.) and by different actors (colleagues, supervisors). A positive link has been found between teachers’ attitudes toward inclusive education and the level of support they perceive (Desombre et al., 2021). Therefore, it is also recommended that schools give teachers necessary materials and adequate work time as well as empathetic feedback.

## Data Availability Statement

The original contributions presented in the study are included in the article/supplementary material, further inquiries can be directed to the corresponding author.

## Ethics Statement

The studies involving human participants were reviewed and approved by the Prince Sattam Bin Abdulaziz University. The patients/participants provided their written informed consent to participate in this study.

## Author Contributions

MA made a substantial, direct, and intellectual contribution to the work, and approved it for publication.

## Conflict of Interest

The author declares that the research was conducted in the absence of any commercial or financial relationships that could be construed as a potential conflict of interest.

## Publisher’s Note

All claims expressed in this article are solely those of the authors and do not necessarily represent those of their affiliated organizations, or those of the publisher, the editors and the reviewers. Any product that may be evaluated in this article, or claim that may be made by its manufacturer, is not guaranteed or endorsed by the publisher.
